# Assessment of Tomato Maturity in Different Layers by Spatially Resolved Spectroscopy

**DOI:** 10.3390/s20247229

**Published:** 2020-12-17

**Authors:** Yuping Huang, Wan Si, Kunjie Chen, Ye Sun

**Affiliations:** 1 College of Mechanical and Electronic Engineering, Nanjing Forestry University, Nanjing 210037, China; huangyuping@njfu.edu.cn (Y.H.); siwan4221@gmail.com (W.S.); 2 College of Engineering, Nanjing Agricultural University, Nanjing 210031, China; kunjiechen@njau.edu.cn

**Keywords:** tomato maturity, different layers, spatially resolved spectra, SVMDA

## Abstract

Tomato maturity is important to determine the fruit shelf life and eating quality. The objective of this research was to evaluate tomato maturity in different layers by using a newly developed spatially resolved spectroscopic system over the spectral region of 550–1650 nm. Thirty spatially resolved spectra were obtained for 600 tomatoes, 100 for each of the six maturity stages (i.e., green, breaker, turning, pink, light red, and red). Support vector machine discriminant analysis (SVMDA) models were first developed for each of individual spatially resolved (SR) spectra to compare the classification results of two sides. The mean spectra of two sides with the same source-detector distances were employed to determine the model performance of different layers. SR combination by averaging all the SR spectra was also subject to comparison with the classification model performance. The results showed large source-detector distances would be helpful for evaluating tomato maturity, and the mean_SR 15 obtained excellent classification results with the total classification accuracy of 98.3%. Moreover, the classification results were distinct for two sides of the probe, which demonstrated even if in the same source-detector distances, the classification results were influenced by the measurement location due to the heterogeneity for tomato. The mean of all SR spectra could only improve the classification results based on the first three mean_SR spectra, but could not obtain the accuracy as good as the following mean_SR spectra. This study demonstrated that spatially resolved spectroscopy has potential for assessing tomato maturity in different layers.

## 1. Introduction

Tomato is the second most consumed vegetable product in the world due to its abundant nutrition, such as sugar, organic acids, and vitamin C [1,2,3]. Maturity is one of the most important indicators to determine the fruit picking time and shelf life. Color is one of the remarkable characteristics closely related to tomato maturity. As tomato matures, the tissue of tomato turns from green to red due to the changes of chlorophyll, anthocyanin and other pigments, along with the changes of the internal texture and chemical composition [4]. Therefore, color should be a significant parameter for assessing tomato maturity.

In recent years, many nondestructive detection techniques, such as magnetic resonance imaging, near-infrared spectroscopy, hyperspectral imaging, machine vision, have been used to assess the maturity or color grade of tomatoes [5,6,7], of which machine vision is the most widely used. Arakeri [8] and Arefi [9] effectively distinguished mature tomatoes from immature tomatoes through machine vision technology, and the classification rates were 96.5% and 96.4%, respectively. Wan et al. [10] used machine vision combined with a back-propagation neural network method to distinguish the three maturity grades (green, orange, and red) of tomato, with an accuracy rate of 99.3%. Although machine vision technology can accurately distinguish the color grade of the fruit surface [11], it is difficult to detect the changes of internal information caused by ripeness. While the visible and near-infrared spectroscopy is capable to obtain information about the internal composition and structure of samples [12,13,14]. Some scholars applied this technology for the determination of tomato maturity [15,16]. Alenazi et al. [17] reported an assessment of fresh tomato fruit at five distinctive maturity stages (breaker, turning, pink, light-red, and red) using the handheld near infrared (NIR) enhanced spectrometer at a wavelength range of 285–1200 nm, and the results showed that the NIR spectroscopy is an effective tool for predicting tomato fruit quality during the harvest stage. Sirisombon et al. [18] used near infrared spectroscopy to identify the three maturity levels (mature green, pink and red) of tomatoes in the wavelength range of 1100–2300 nm, with an optimal classification accuracy rate of 96.9%. Clément et al. [19] established a mathematical model for the color parameters of tomatoes using visible and near infrared spectroscopy (400–1500 nm) with the correlation of 95%. However, conventional visible and near-infrared spectroscopy is a single-point or specific area measurement, which could not provide spatial information of the research object. Thus, for such objectives with heterogeneous structure like tomatoes, conventional visible and near-infrared spectroscopy could not accurately determine their quality.

**Figure 1:**
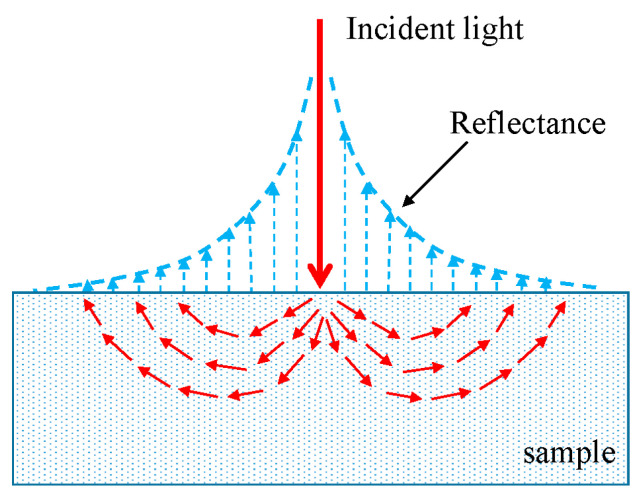
Spatially resolved spectra at different source-detector distances.

Spatially-resolved spectroscopy is an emerging spectroscopic technique for measuring reflectance from the sample at different spatial distances between a point light source and detectors. Spatially-resolved (SR) spectra contain information about the sample at different layers (or depths) since the transport path of photons generally forms a “banana-shape” in biological tissues [20], shown in Figure 1. Short spatial distances between the illumination and detectors would provide top layer information of the sample, while large source-detector distances is useful to obtain deep layer information of the sample [21]. Numerous studies reported on assessment of quality attributes using spatially resolves spectroscopy with different orientations [4,22,23]. Meanwhile, the limited spatial distances and wavelength range could not provide adequate information about samples. Huang et al. [21] first developed a new spatially resolved spectroscopic system for property and quality assessment of horticultural and food products with source-detector distance of 1.5–36 mm. Then, spatially resolved spectroscopic system was applied to predict the soluble solid content (SSC), pH, and firmness of tomato over the spectral range of 550–1650 nm, these results demonstrated that the spatially resolved spectroscopy has advantages over conventional spectroscopy for enhancing quality assessment of tomatoes [24,25]. However, most of the previous studies were focused on a single quality parameter, whereas maturity is a comprehensive quality that contains a variety of quality information, and there are still many challenges that must be addressed for its application to assess tomatoes maturity. Moreover, tomato ripening is a non-uniform process, on the one hand, the color distributed variously with space during the ripeness, on the other hand, chemical constituent and texture also changed with interspace as tomato ripeness, thus single surface color indexes may not provide adequate information for tomato maturity evaluation. Since the acquisition probe of the spatially resolved spectroscopic system includes multiple fibers, it can obtain internal information at different depths about the sample within a certain detection distance. Thus, the spatially resolved spectroscopic system should be more suitable for evaluating the tomato maturity.

Therefore, in this study, the feasibility of spatially resolved spectroscopic system for assessing tomato maturity in different layers was analyzed and discussed. The specific objectives of this study were to: (1) acquire SR spectra of each tomato sample in different layers over the spectral range of 550–1650 nm; (2) reveal the relationship between quality attributes and maturity stages; (3) compare the classification results for tomato maturity in different layers; (4) analyze the classification results with various locations in the same layer.

## 2. Material and Methods

### 2.1. Samples

A total of 600 ‘Sun Bright’ tomato samples at six maturity stages were picked from a research field at Michigan State University’s Horticultural Research and Teaching Center (Holt, MI, USA). The samples were cleaned and inspected to make sure there were no external defects for tomato samples. The fruit were then classified into six maturity stages (i.e., Green, Breaker, Turning, Pink, Light-Red and Red) based on the USDA standards [26]. Each stage had 100 tomato samples. Before the experiment started, tomato samples were kept at room temperature (about 25 °C) for at least 12 h to make sure each of the whole samples had consistent temperature to avoid the temperature influence for spectra or analysis results. Spectral acquisitions were first taken for each of sample using the spatially resolved spectroscopic system over the spectral range of 550–1650 nm. Thereafter, the samples were subjected to physical and chemical experiment.

### 2.2. Spatially Resolved (SR) Spectra Measurement

**Figure 2:**
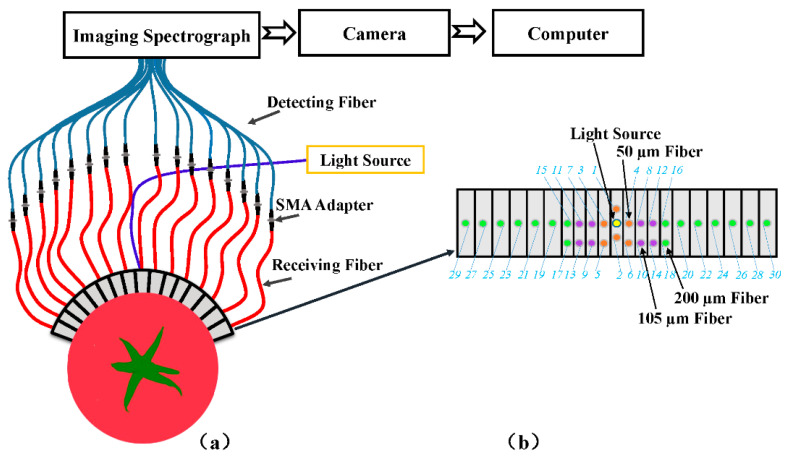
Schematic of the spatially resolved spectroscopic system (**a**), and the acquisition probe (**b**).

Thirty spatially resolved spectra for each of tomato samples were acquired using a newly developed spatially resolved spectroscopic system (Model 1003B-10152, Headwall Photonics, Inc., Fitchburg, MA, USA), shown as Figure 2, with the advantages of large source-detector distances of 1.5–36 mm, flexibility for samples of flat or curved surface, and enhanced dynamic range by using different sized fibers. A detailed description of the acquisition probe was given by Huang [21]. During the spectral collection, the spatially resolved spectroscopic system was set with an integration time of 60 ms and the light source output power was set for 240 W. The acquisition probe was directly contact with the equatorial area of tomato fruit. The symmetric pair spectra with the same source-detector distance were performed to average in order to assess the influence of source-detector distances for tomato maturity detection, resulting in 15 mean_SR spectra with different source-detector distances. A white cylindrical Teflon block was used as a white reference, and the same experimental procedure was taken for obtaining white reference spectra to calibrate the sample spectra. Whereas the dark reference spectra were acquired by closing the light source in a dark room. The relative spatially resolved spectra for the samples were calculated using the white, the dark and the original sample spectra [27]. The relative reflectance spectra were used in further data analysis.

### 2.3. Quality Analysis of Tomato Fruit

After spectral acquisition, nondestructive and destructive firmness detection, soluble solid content (SSC) test, and pH measurement were performed on the tomato samples. Acoustic firmness of each samples was obtained by a desktop acoustic firmness sensor (AFS, AWETA, Nootdorp, The Netherlands), while the other two firmness parameters, the compression slope and the puncture maximum force, were subjected by a Texture Analyzer (Model TA.XT2i, Stable Micro System, Inc., Surrey, UK). The SSC value for each of the tomatoes was acquired from a handheld digital refractometer (model PR-101, Atago Co., Tokyo, Japan), and pH value for each of tomato samples was carried out using a portable pH meter (model HI98161, Hanna Instruments, Inc., Woonsocket, RI, USA). The analysis of variance (ANOVA) was performed on the quality parameters (AF, CS, PA, SSC, and PH) of six maturities of tomatoes using SPSS 18.0 statistics software. The level of *p* < 0.05 was considered significant in all analyses.

### 2.4. Tomato Maturity Classification Models

Since the maturity was determined by many indicators, such as chemical composition, texture and color, the obtained spectra may not present linear relation with tomato maturity. Support vector machine discriminant analysis (SVMDA), as commonly used nonlinear model, uses LIBSVM package to calculate the probabilities of each sample belonging to each possible class. This method first calculates one-against-one pairwise class probabilities as a function of decision value, using the training data. The class probabilities are then found as an optimization problem using these *k ×* (*k* − 1)/2 pairwise probabilities (assuming there are *k* classes). In this study, SVMDA models were first developed for each of the 30 individual SR spectra to compare the classification results for two sides of acquisition probe so that it could provide some foundation for evaluating symmetry of tomato maturity. Moreover, in order to assess the tomato maturity on different layers, 15 mean_SR spectra with different source-detector distances and SR combination by averaging all SR spectra were also used to establish the SVMDA models.

The 600 tomato samples were randomly divided into training and test sets. There were 360 and 240 samples in training and test sets, respectively. SVMDA models were developed using MATLAB R2017a (The MathWorks, Inc., Natick, MA, USA) combined with PLS Toolbox 8.2 (Eigenvector Research, Inc., Wenatchee, WA, USA). Autoscale is an exceptionally common preprocessing method which uses mean-centering followed by division of each column (variable) by the standard deviation of that column. It is a valid approach to correct different variable scaling and units if the predominant source of variance in each variable is signal rather than noise. Therefore, autoscale preprocessing was performed in each of individual SR spectra. Venetian blinds cross-validation is suitable for the data that are already in random order [28] and was used in the training set to calibrate the models. The performance of the models was evaluated by classification accuracy (recognition rate), sensitivity, and specificity for the test set of samples.

## 3. Results and Discussion

### 3.1. Differences of Quality Attributes for Tomatoes at Six Maturity Stages

Table 1 shows the means and standard deviations of the quality parameters, and ANOVA for the quality parameters of tomatoes in six maturity stages using SPSS 18.0 statistics software. The SSC and pH showed an upward trend, which meant that the sugar content accumulated and the acidity content decreased as tomato matures from green to red. While the firmness parameters like acoustic firmness (AF), compression slope (CS), and puncture maximum force (PF) showed a downward trend, which indicated that as tomato fruit turns green to red, the firmness became soft, which is also agreement with previous studies [4]. All of the quality parameters showed significant differences between different maturity stages excluding acoustic firmness, which could be partly because the tomato in breaker, turning and pink stages, the color began to change, the acoustic test measured the global properties of tomato sample and the resonant frequency in these stages may have limited changes, resulting in no significant differences of these three maturity stages. Considering the results, tomato maturity was influenced by not only chemical composition, such as SSC and pH, but also structural characteristics like firmness.

### 3.2. Spectral Features

**Figure 3:**
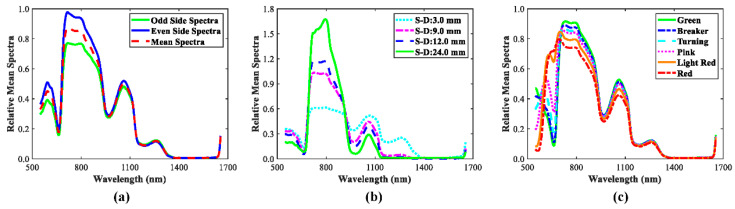
Mean reflectance spectra of tomatoes at turning stage for odd and even sides with source-detector distance of 6.0 mm and the mean spectra of these two spectra (**a**), the mean spectra with different source-detector distances for tomatoes at breaker stage (**b**), and the mean spectra at six maturity stages with the source-detector distance of 6.0 mm (**c**).

**Figure 4:**
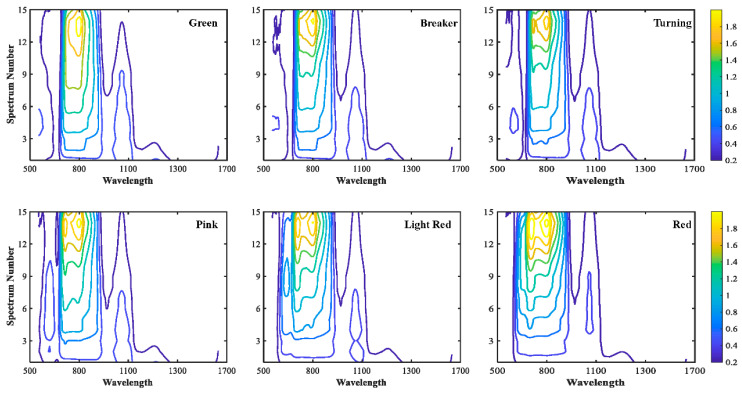
The contour maps for 15 SR spectra for tomato at six maturity stages over the spectral region of 550–1650 nm.

Figure 3a shows the SR spectra at source-detector distance of 6.0 mm in two sides of the probe and the mean spectra of two sides. The overall pattern for SR spectra in two sides was similar excluding in the spectral range of 700–900 nm, which suggested there should be some discrepancies for the classification results of these two SR spectra, this speculation was demonstrated in next section. Four mean_SR spectra at different source-detector distances (3 mm, 9 mm, 12 mm, and 24 mm) were illustrated in Figure 3b. Large differences in the pattern of these spectra were observed over the whole spectral range, because these spectra contained information at different tissue layers (or depths) of the sample. It suggested each SR spectra would have different classification capabilities for tomato maturity, and therefore it is important that SVMDA models should be developed for each mean_SR spectra so as to determine the best source-detector distance for tomato maturity detection. In the near infrared region, the spectra were influenced by water absorption bonds and C-H bonds at 970 nm, 1180 nm, and the spectral beyond 1400 nm. Figure 3c displays noticeable differences in relative reflectance around 560 nm and 680 nm for six maturity stages, which could be due to the fact that as tomato turned green to red, anthocyanin (at 560 nm) increased along with chlorophylls (at 680 nm) decreased [24,29,30]. Figure 4 shows the contour maps for 15 mean_SR spectra for tomatoes at six maturity stages. The prominent differences were inspected from contour maps for six tomato maturity stages, particularly in the spectral range of 550–1100 nm. In addition, extremely weak intensity appeared in the spectral range around or beyond 1300 nm due to the high absorption bonds of O-H. Overall, combined with mean_SR spectra at different source-detector distances could provide a fingerprint for each of tomato maturity.

### 3.3. Discrimination Models for Tomato Maturity

**Figure 5:**
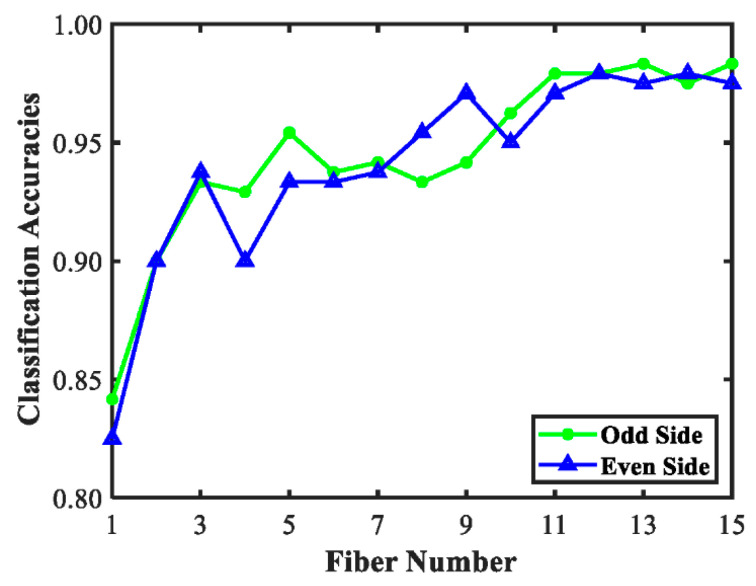
The total classification accuracies for recognition tomato maturity based on 15 individual SR spectra on two sides.

Figure 5 illustrates the total classification accuracies for identification of tomato maturity using 30 individual SR spectra on two sides of acquisition probe. The classification results generally presented upward trend, which indicated that the SR spectra with a relatively large source-detector distance would have better recognition results for tomato maturity. Moreover, even if the SR spectra with the same source-detector distance on two sides, the obtained results were distinct, especially for the middle SR spectra with source-detector distances of 6.0–12.5 nm. It suggested the structure and chemical composition distributed variously in the different positions of tomato tissue. Previous study reported the source-detector distance was similar to the light penetration depths for the sample [31], which meant when the light penetration depths about 6.0–12.5 nm of the tomato fruit, the single-point detection could not be accurate due to the relative large distinct results. The classification results for each individual SR spectra on two sides of the acquisition probe demonstrated that, even in the same layer for detection, the recognition for tomato maturity still had diversities due to the heterogeneity of tomato tissue. This suggested the single-point or small-area detection could not be suitable for assessing non-uniform tissue in fruit, such as tomato fruit.

Table 2 summarizes the tomato classification results in training and test sets based on SVMDA models using the mean_SR spectra of two sides and the mean spectra of all spectra, to evaluate the classification results for tomato maturity in different layers and compare with the results for the combination of all layers. The recognition results in calibration set were consistently better than both cross-validation and test sets. Moreover, as the spectrum number increased, the calibration results become better and stable, specifically for the SR spectra after mean_SR 7, each of the mean_SR spectra could reach the best classification accuracies of 100%. The classification in cross-validation had relative consistent results except first two mean_SR spectra. Good classification results in cross-validation indicated the established models had reliable classification performance. In test set, the overall trends for classification accuracies was ascendant along with the spectrum number increased with differences of 18.6% between the optimal and worst spectra. Moreover, when the spectrum number reached mean_SR spectra 9, the classification results could consistently achieve above 95% for the following spectra, indicating large source-detector distances were useful for maturity evaluation. Furthermore, as shown in Table 1, tomato maturity stages were related to the chemical compositions and structural characteristics, light absorption is related to the chemical composition, while the scattering depends on the structural characteristics. The obtained SR spectra contained the compounding effect of absorption and scattering, thus the SR spectra with large source-detector distances had a long photon path and strong scattering and absorption in the sample, which would reveal more information about sample, resulting in better classification results for tomato maturity. In addition, there were large differences for the first three mean_SR spectra, and the results were relative lower, which could be due to the limited information about samples at top layers. Mean_SR 6 with source-detector distance of 9.0 mm had relative higher results, however, the classification accuracies decreased as the spectrum number increased until mean_SR 9. This suggested the SR spectra with relative short spatial distances between light source and detector could not give stable classification results. The SR combination by averaging all the SR spectra, named mean_all_SR spectra, was also presented in Table 2, its classification result was only better than the first three mean_SR spectra, indicating that averaging all SR spectra could not be an effective means for improvement of classification results. It also suggested that, with the mean of all data, it is possible to enlarge some irrelevant variables for model building.

Further analysis is shown in Table 3 to summarize classification results for optimal mean_SR spectra and mean_all_SR spectra of tomatoes at six maturity stages. The mean_SR 15 presented the best recognition rates for six maturity stages with the value of 100% in the training set, besides, the optimal recognition rates also showed in test set for green, pink, and light red stages. Breaker stage had relative lower classification accuracy due to two samples misclassification, while in turning and red stages, only one sample misclassified in neighboring stage. Mean_all_SR spectra had the same recognition results with mean_SR 15 in green and breaker stages, but the recognition rate only reached 75% in turning stage with ten samples misclassification in neighboring stages, while mean_SR 15 could present 30% improvement for accuracy with the value of 97.5%. Compared with the mean_SR 15 spectra, mean_all_SR spectra gave lower accuracies for the following stages with differences of 5.3% for pink stage, 12.2% for light red stage and 2.9% for red stage, which demonstrated mean_all_SR spectra did not have prominent superiority for assessing tomato maturity.

Sensitivity and specificity were two important parameters for assessing the performance of SVMDA models. The definition of the sensitivity is the proportion of true positives that are correctly identified, while specificity is defined as the proportion of true negatives that are correctly identified. Further breakdowns of sensitivity and specificity at six maturity stages using mean_SR 15 and mean_all_SR spectra were presented in Table 4. Mean_SR 15 had consistently higher values for both sensitivity and specificity than that of mean_all_SR spectra except green stage, indicating better model performance for mean _SR 15. In green stage, it is interesting that mean_all_SR spectra had excellent results for both sensitivity and specificity with the value of 1.000, which suggested mean_all_SR spectra had ascendancy in assessment of green tomatoes. In addition, for both of two spectra, the values of specificity in six maturity stages were more stable than sensitivities, specifically for mean_SR 15 with only 0.5% difference between optimal and worst values, which demonstrated the models had reliable performance for tomato maturity classification.

### 3.4. Feature Extraction

**Figure 6:**
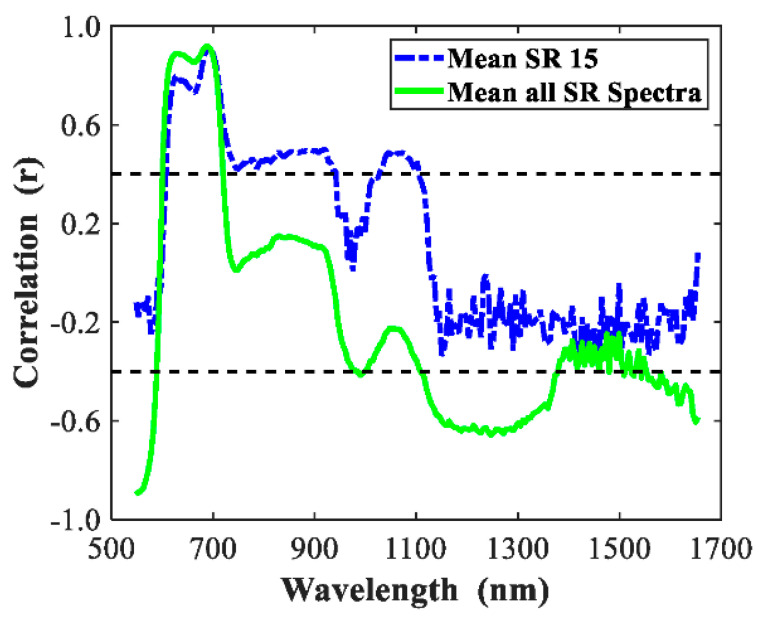
Correlation curves for tomato between six maturity stages and relative reflectance of individual wavelengths for the spectral region of 550–1650 nm.

Correlation curve between the maturity stages and single wavelengths of the visible and near-infrared (Vis/NIR) spectra is presented in Figure 6. The spectral correlations of mean_SR 15 and mean_all_SR_Spectra lower than 0.4 were removed to extract these variables that had high correlation with wavelengths, which would obtain two new spectra. The classification results for these two new spectra were shown in Table 5. Compared with the full wavelength spectra, new mean_SR 15 had relatively poor results in both the training and test sets, which suggested each of the variables for mean_SR 15 spectra provided a contribution of model building. While for new mean_all_SR spectra, after removing these variables that had low correlation with wavelengths, the classification results became better in training set, and the classification results were the same in test set except turning stage with a bit change, which demonstrated that variable selection was effective for mean_all_SR_Spectra.

### 3.5. Discussion

This research demonstrated that the source-detector distance had large influence on tomato maturity classification. The classification accuracies overall raised as the source-detector distance increased, and these SR spectra at source-detector distance of 16.0–36.0 mm overall gave more consistent classification results. For these SR spectra with a large source-detector distance, the transport path of photons become longer, the more interactions of photons with objects, resulting in more absorption and scattering in the sample tissue [32]. Quality parameters like SSC, pH and firmness were directly related to the maturity, which is, in turn, influenced by the pigment contents of the fruit tissue. As indicated above, light absorption is the annihilation of a photon by matter, which depends on the chemical composition of the medium, while scattering of photons is a physical process that largely depends on the structural properties of the sample. This may explain the SR spectra with large source-detector distances had relative higher classification results. Moreover, the SR spectra at the same source-detector distances with varied positions showed distinct results. Tomato fruit is heterogeneous, consisting of an outer wall of pericarp with a dense tissue structure, followed by locular cavities filled by a clear substance containing the seeds. During the ripening process, the changes of chemical constituents, structural properties, and pigments, as well as the changes of cell walls and pectin solubilisation resulted in an ununiformed composition and texture [1], which could be possible to explain the various results for different position in the same detection layer. Furthermore, the combination of SR spectra, which is the mean of all SR spectra, could not provide enhanced results, which suggested each of individual SR spectra contained interference information or irrelevant variables, after averaging all SR spectra, feature information may decrease, and introduce more interference information or irrelevant variables, leading to unsatisfying results.

The classification results in this study were also comparable or better with previous studies using near infrared spectroscopy. Sirisomboon et al. [18] obtained classification results of 96.85% and 100% for mature green and red tomatoes, respectively, Tiwari et al. [33] correctly predicted 71% of immature and 85% of mature green tomatoes and Clément [34] predicted the tomato maturity stage with a coefficient of determination of 0.93. Accurate comparison of the results obtained by spatially resolved spectroscopic system in this research with previous studies can be difficult, because many factors (e.g., growth condition, variety, methods used for sampling and data processing, etc.) can influence the classification results. From the results in Table 2 and Table 3, the classification results of the six maturity stages of tomato by the spatially resolved spectroscopic system basically exceeds 90%, which demonstrated this technology is effective for classifying tomato maturity stages. Moreover, a follow-up study should be considered to further improve the classification results, such as further optimization of the data (i.e., feature extraction, variable selection, advanced data preprocessing, and modeling techniques).

## 4. Conclusions

The paper reported on assessment of tomato maturity in different layers using spatially resolved spectroscopy over the wavelength range of 550–1650 nm. Thirty individual SR spectra were used to establish the SVMDA models and the classification results for two sides of an acquisition probe were compared. Mean_SR spectra were adopted to evaluate the influence of source-detector distances for maturity classification. Moreover, the mean of 30 individual SR spectra was also applied to analyze and compare the performance of the models. The classification results indicated that tomato maturity classifications were influenced by the source-detector distances as well as the detection location of the tomato. The mean of all SR spectra could not achieve satisfactory results, which demonstrated that more information about samples sometimes could not obtain improved results. The mean_SR 15 acquired the optimal classification results for tomato maturity with recognition rates of 100%, 94.6%, 97.5%, 100%, 100%, and 97.1% for green, breaker, turning, pink, light red, and red stages. In this study, spatially resolved spectroscopy was employed to assess the feasibility for tomato maturity measurement in different layers.

## Figures and Tables

**Table 1:** Means and standard deviations of the quality parameters for tomatoes with different maturities *.

Maturity	AF	CS	PF	SSC	pH
Green	9.03 ± 1.88 ^a^	34.57 ± 7.30 ^a^	17.52 ± 2.13 ^a^	4.42 ± 0.34 ^a^	3.98 ± 0.22 ^a^
Breaker	5.75 ± 1.33 ^b^	18.95 ± 3.86 ^b^	15.08 ± 2.16 ^b^	4.61 ± 0.43 ^b^	4.02 ± 0.10 ^b^
Turning	5.54 ± 1.37 ^b^	16.04 ± 3.31 ^c^	13.89 ± 2.16 ^c^	4.80 ± 0.47 ^c^	4.10 ± 0.10 ^c^
Pink	5.38 ± 1.48 ^b^	13.91 ± 2.78 ^d^	11.69 ± 1.73 ^d^	5.12 ± 0.44 ^d^	4.14 ± 0.09 ^d^
Light Red	4.66 ± 1.36 ^c^	10.54 ± 1.67 ^e^	8.72 ± 1.46 ^e^	5.37 ± 0.50 ^e^	4.24 ± 0.11 ^e^
Red	4.04 ± 1.36 ^d^	8.20 ± 2.17 ^f^	7.12 ± 1.54 ^f^	5.73 ± 0.56 ^f^	4.34 ± 0.12 ^f^

* Numbers for the same columns with different letters (a, b, c, d, e, f) are different at the level of 0.05 based on the analysis of variance.

**Table 2:** Total classification accuracies for tomato maturity by using SVMDA for 15 mean_SR spectra and mean spectra of all SR spectra ^a^.

Type of Spectra	Training Set		Test Set
	Cal (%)	CV (%)	Pred (%)
Mean_SR 1	92.5	85.8	83.3
Mean_SR 2	94.7	90.6	82.9
Mean_SR 3	98.9	93.9	90.0
Mean_SR 4	100	96.4	91.3
Mean_SR 5	98.9	95.0	92.9
Mean_SR 6	100	95.3	96.7
Mean_SR 7	98.9	95.8	93.8
Mean_SR 8	100	95.0	93.8
Mean_SR 9	100	93.9	95.4
Mean_SR 10	100	98.1	97.9
Mean_SR 11	100	97.2	97.5
Mean_SR 12	100	99.2	97.5
Mean_SR 13	100	97.2	95.4
Mean_SR 14	100	96.1	97.1
Mean_SR 15	100	96.9	98.3
Mean_all_SR Spectra	98.6	93.1	91.3

^a^ Cal: calibration; CV: cross-validation; Pred: prediction.

**Table 3:** Classification results for the six maturity of tomatoes by using Support Vector Machine discriminant analysis for optimal mean_SR spectra (mean_SR 15) and mean spectra of all SR spectra ^a^_._

Type of Spectra	Maturity	Training Set/%	Test Set/%						
			G	B	T	P	L	R	Accuracy
Mean_SR 15	G	100	42	1	0	0	0	0	100
	B	100	0	35	1	0	0	0	94.6
	T	100	0	0	39	0	0	0	97.5
	P	100	0	1	0	40	0	0	100
	L	100	0	0	0	0	46	1	100
	R	100	0	0	0	0	0	34	97.1
Mean_all_SR_Spectra	G	98.3	42	0	0	0	0	0	100
	B	98.4	0	35	5	0	0	0	94.6
	T	96.7	0	2	30	2	0	0	75.0
	P	98.3	0	0	5	38	0	1	95.0
	L	100	0	0	0	0	41	1	89.1
	R	100	0	0	0	0	5	33	94.3

^a^ G: green; B: breaker; T: Turning; P: Pink; L: light red; R: red.

**Table 4:** Performance of the SVMDA models developed by optimal mean_SR spectra and mean all SR spectra ^a^.

Parameters	Spectral Type	G	B	T	P	L	R
Sensitivity	Mean_SR 15	1.000	0.946	0.975	1.000	1.000	0.971
	Mean_All_SR_Spectra	1.000	0.946	0.750	0.950	0.891	0.943
Specificity	Mean_SR 15	0.995	0.995	1.000	0.995	0.995	1.000
	Mean_All_SR_Spectra	1.000	0.975	0.980	0.970	0.995	0.976

^a^ G: green; B: breaker; T: Turning; P: Pink; L: light red; R: red.

**Table 5:** Classification results for the six maturity of tomatoes by using Support Vector Machine discriminant analysis for new mean_SR 15 and new mean_all_SR spectra.

Type of Spectra	Maturity	Training Set/%	Test Set/%						
			G	B	T	P	L	R	Accuracy
New_mean_SR 15	G	98.3	40	5	0	0	0	0	95.2
	B	88.9	2	28	0	0	0	0	75.7
	T	88.3	0	3	33	4	0	0	82.5
	P	86.7	0	1	7	35	2	1	87.5
	L	94.4	0	0	0	1	34	4	73.9
	R	98.4	0	0	0	0	10	30	85.7
New_mean_all_SR_Spectra	G	100	42	1	0	0	0	0	100
	B	98.4	0	35	5	0	0	0	94.6
	T	96.7	0	1	29	2	0	0	72.5
	P	100	0	0	5	38	1	1	95.0
	L	100	0	0	1	0	41	1	89.1
	R	100	0	0	0	0	4	33	94.3
